# Heat Transfer Measurements with Surface Mounted Foil-Sensors in an Active Mode: A Comprehensive Review and a New Design

**DOI:** 10.3390/s90403011

**Published:** 2009-04-23

**Authors:** Horst Mocikat, Heinz Herwig

**Affiliations:** Hamburg University of Technology, Institute of Thermo-Fluid Dynamics, Denickestr. 17, D-21073 Hamburg, Germany; E-mail: horst.mocikat@mb.tu-chemnitz.de

**Keywords:** Double Foil Sensor, Active Mode, Passive Mode, Calibration Procedures, Convective Heat Transfer

## Abstract

A comprehensive review of film-sensors shows that they are primarily operated in a passive mode, i.e. without being actively heated to an extent, whereby they create a heat transfer situation on their own. Only when these sensors are used for wall shear stress measurements, the detection of laminar/turbulent transition, or the measurement of certain flow velocities, they are operated in an active mode, i.e. heated by an electrical current (after an appropriate calibration). In our study we demonstrate how these *R(T)*-based sensors (temperature dependence of the electrical resistance *R*) can also be applied in an active mode for heat transfer measurements. These measurements can be made on cold, unheated bodies, provided certain requirements with respect to the flow field are fulfilled. Our new sensors are laminated nickel- and polyimide-foils manufactured with a special technology, which is also described in detail.

## 1. Introduction

Sensors made of thin metal films can be found in a wide variety of forms and sizes and are used for various purposes. Their main field of application is the direct measurement of temperatures and heat flux density in heat transfer problems from which heat transfer coefficients *h* or their nondimensional counter part, the Nusselt numbers Nu, can be determined. They are, however, also used for the “indirect” measurement of wall shear stress, τ_W_, the detection of laminar/turbulent transition, or the measurement of certain flow velocities.

Typical examples of wall temperature sensors are reported in [1–5], heat flux sensors in [6–9], wall shear stress sensors in [10–17], transition detection in [18–20], and flow velocity measurements in [21–26]. Instead of compiling a large number of studies we try to characterize typical features of this kind of sensors and then set up a scheme by which these sensors can be classified.

The common feature of the sensors under consideration is that their essential part consists of one, two or more thin metal *films* or *foils*. Here, and from now on,

- *films* are very thin metal layers. They are so thin (typically 10 – 300 nm) that their physical properties and especially their temperature coefficient of electrical resistance, *α*, deviate from the corresponding bulk values [22,27]. Thin film sensors typically are manufactured by a sputtering technique (metal deposition on a substrate) [1,22].
- *foils* are thin metal layers typically of thickness ≤ 2 μm. They are thick enough for their physical properties to be the same as those of the corresponding bulk material. Thin foil sensors can be made from sheets of metal foils, glued to a carrier foil and cut or etched in its final shape. This is our new “laminating and etching technology” described in Chapter 4.

Alternatively sensors can be designed and fabricated using MEMS technology. These sensors consist of Si-wafers containing the sensitive structures or elements, respectively. One of the most important benefits then is the possibility of miniaturization [13,14,24].

In all sensors advantage is taken of the temperature dependent electrical resistance *R = R(T)* of the thin metal films or foils in one or the other way. Therefore these sensors are also called *resistance temperature sensor RTS*.

Note that a sensor as a technical instrument (commercially available) consists of sensitive elements and otherwise structural elements, such as connectors, substrates, protective covers (layers, films) and so on. With regard to different sensor properties a sensitive element only or the sensor as a whole must be considered. Therefore we distinguish between the terms “temperature sensitive element, TSE” and “sensor, S”, or “resistance temperature sensor, RTS”, respectively.

## 2. Characterization Scheme for RTS Film- and Foil-Sensors

In order to characterize RTS film- and foil-sensors with respect to their most important features, two categories are introduced:

❒ Their *basic geometrical structure*, determined by the quantity to be measured, which can be
  - wall temperature *T*_W_
  - wall heat flux density *q̇*_W_
  - wall shear stress τ_W_
  - location of laminar/turbulent transition (*l/t*)
  - flow velocity, individually defined (*u*)
❒ Their *application form* on the wall, determined by the mode of measurement, which can be
  - a passive mode (negligible self heating)
  - an active mode (strong self heating)

Here negligible self heating in the passive mode means that the electrical current is so weak that no detectable dissipation occurs. In this mode each film or foil can only act as a *temperature sensitive element* (TSE). In the active mode, however, a strong electrical current creates a heat transfer situation by its own, i.e. the dissipated electrical energy is transferred into the fluid as a wall heat flux, characterized by its density *q̇*_W_, provided the metal film or foil is thermally isolated against the wall and radiative heat transfer can be neglected. In this mode a film or foil is able to act as a temperature sensitive element (TSE) as well as a *heating element*, HE. Due to the double mode (TSE and HE) the films or foils will all be called *sensitive elements*, SE.

**Figure 1.**
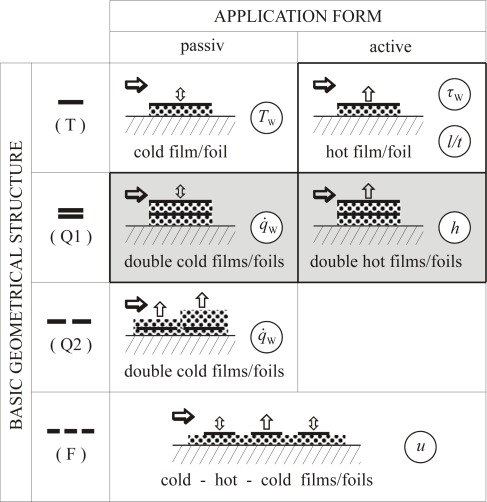
Characterization scheme for RTS film- and foil-sensors. 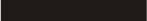: metal film/foil; 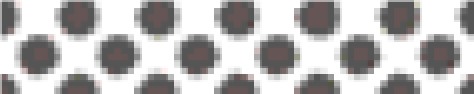: solation,thermal barrier (e.g., polyimide foil); 
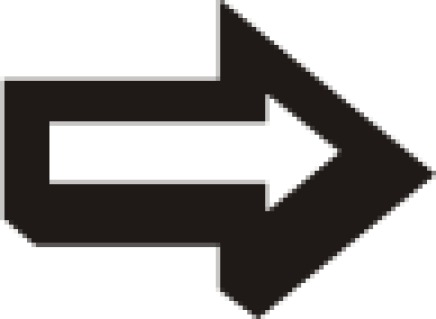
: flow direction; 
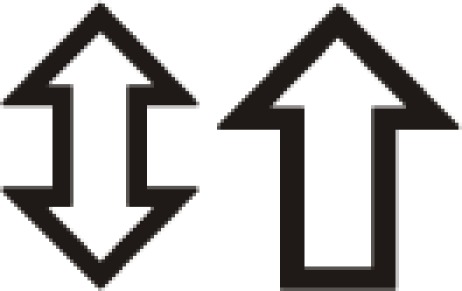
: heat flux directions; 
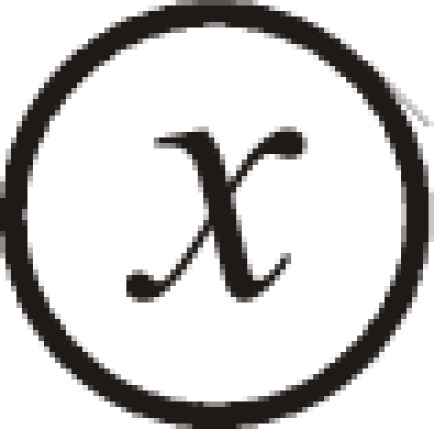
: targeted quantity, *x = T*_W_*, τ*_W_*, …*

In Figure 1 four *basic geometrical structures* are shown in their two *application forms*, passive and active. They are:

T: one single film/foil for temperature measurement
Q1: two single films/foils, one upon the other, for heat flux measurements
Q2: two single films/foils, side by side, for heat flux measurements
F: two or more single films/foils, side by side, for flow measurements

The metal films or foils schematically shown in Figure 1 represent the SE (sensitive elements). Basically they are straight electrical conductors with length *l*, width *b*, thickness *d* and thus with an area of the sensitive element *A*_SE_ *= bl* and a cross section *A_c_* = *bd*. Its temperature dependent resistance *R(T)* is given by:

(1) $$R\left(T\right)=\rho_{e}\left(T\right)\frac{l}{\mathrm{bd}}$$

with the specific electrical resistivity:

(2) $$\rho_{e}\left(T\right)=\rho_{e0}\left[1+\alpha \left(T-T_{r}\right)\right]$$

Here *α* is the temperature coefficient of the electrical resistivity, with the temperature of the element, *T*, measured in [°C] and a reference temperature *T*_r_ = 0 °C. The linear temperature function (2) is a good approximation for most metals in moderate temperature ranges. From (1) and (2) the resistance

(3) $$R\left(T\right)=R_{0}\left[1+\alpha \left(T-T_{r}\right)\right]$$

and from (3) the temperature sensitivity of the SE acting as TSE follows:

(4) $$S_{R}\equiv \frac{\partial R}{\partial T}={\alpha R}_{0}$$

For temperature measurements, as well as for heating, there must be a known voltage *U* along the electrical resistance *R* which here is the sensitive element (SE). From this resistance the power:

(5) $$P=U^{2}/R=\dot{Q}$$

is transferred to the fluid as heat. With the *R(T)* function according to (3) the temperature *T* is:

(6) $$T=\frac{R\left(T\right)-R_{0}}{\alpha R_{0}}$$

and thus immideately follows from the measurement of *U*. As a consequence the relative uncertainty of *U*, *ΔU / U*, is directly linked to the relative uncertainty of *T*, i.e. *ΔT / T*. This will be further accounted for in Section 7.

There are three important aspects regarding the design and the manufacturing of the sensors, in particular the SE with respect to the measurement objectives:

- Accurate measurements require high values of sensitivity *S_R_*. The coefficient *α* is given by the sensitive material, influenced by the manufacturing technology of the SE due to different film or foil thicknesses. The resistance *R*_0_ is given by its material characteristic value *ρ_e_* and by its geometrical dimensions. According to (4) and (5) high values of resistance *R* are advantegeous to achieve high sensor signals *U* with low uncertainties Δ*U*.
- Local measurements require small sensor areas *A*_SE_, i.e. small *l* and *b* for three dimensional and small *b* for two dimensional cases. Characteristic length scales of the phenomena under consideration may vary over a wide range or even orders of magnitude. The geometric sensor dimensions must match these scales. In order to get high values of resistance, however, the sensor should be as large as possible.
- Time resolved measurements require small response times. These time constants are characteristic not only of the TSE but also of the complete sensor on the body under observation. A complete sensor consists of one or more SE, the substrate or carrier on which the SE are mounted, glued or deposited and a casing if requiered. In order to get small response times the sensor should be as small as possible, especially with respect to its heat capacity. The SE should be in good thermal contact with the object under consideration. In the case of wall temperature measurement ((T) passive in Figure 1) a good thermal contact to the body must be guaranteed. For wall shear stress measuremens with a hot-film ((T) active in Figure 1) a good thermal contact to the fluid and a good thermal insulation against the body are requiered.

In Figure 1 various sensor designs are shown. The grey shaded fields mark the kind of sensor which we want to present here in detail. This sensor type (Q1) will be called “Double Foil Sensor (DFS)” in the following. In its passive mode it is a conventional sensor for heat flux density and wall temperature measurements. In its active mode it is a double hot foil sensor by which the heat transfer coefficient *h* can be determined, once it is calibrated for this purpose in a special calibration procedure. This calibration is crucial, it is described in Chapter 5, below.

The new features of our sensor will be demonstrated by comparing its application forms “passive and active mode”. In a convective heat transfer situation the DFS in its passive mode can be used to determine *q̇*_W_ and from that the heat transfer coefficient *h*. The active DFS produces a heat transfer situation by itself and its measured values result in a sensor specific heat transfer coefficient *ĥ*. After a special calibration process, a relation *h*(*ĥ*) can be determind. Therefore *h* is the targeted quantity as shown in Figure 1. Experimental results are described and discussed in chapter 6 below.

## 3. The Heat Transfer Sensor Concept

**Figure 2.**
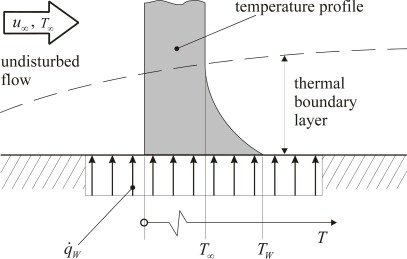
Part of a geometry with heat transfer to the adjacent fluid.

Our double foil sensor DFS of Q1-type in Figure 1 can be used in the passive and the active mode. In the passive mode heat transfer can be in both directions, whereas in the active mode it is always from the wall to the fluid. This situation is depicted in Figure 2, where a typical heat transfer situation is shown which quantitatively is described by a local heat transfer coefficient:

(7) $$h=\frac{{\dot{q}}_{W}}{T_{W}-T_{\infty }}$$

or the equivalent non dimensional Nusselt number:

(8) $$\text{Nu}=\frac{\mathrm{hL}}{k}=\frac{{\dot{q}}_{W}L}{k\left(T_{W}-T_{\infty }\right)}$$

Here *q̇*_W_ is the heat flux density at the wall measured in W/m^2^, *T*_W_ and *T*_∞_ are the wall and ambient temperatures, respectively, *k* is the thermal conductivity of the fluid and *L* is a characteristic length of the geometry on which sensors are mounted.

In order to determine the local values of *h* or Nu three quantities must be measured:

- the local heat flux density, *q̇*_W_
- the local wall temperature, *T*_W_
- the ambient temperature, *T*_∞_

As long as *q̇*_W_ does not affect the flow field, *h* and Nu do not depend on *q̇*_W_, since a changed heat flux density *Cq̇*_W_ with *C* ≠ 1 leads to a temperature difference *C*(*T*_W_ – *T*_∞_) and thus to an unchanged *h* or Nu. This will be the case for forced convection when the temperature dependence of physical properties has only a negligible effect.

Furthermore, as long as the flow is fully turbulent, *h* and Nu are almost independent of the “temperature history” of the oncoming flow, since in these flow situations the physics of heat transfer are known to be local in character.

Taking these two aspects together one can conclude that the local heat transfer situation on a globally and strongly heated geometry is equivalent to that on a locally and marginally heated part of the geometry. This exactly is the basic idea behind our sensor with a calibration function *h*(*ĥ*), correlating the sensor specific heat transfer coefficient *ĥ* and that of the flow field *h*. Instead of measuring *q̇*_W_, *T*_W_ and *T*_∞_ on a body under full thermal load in order to get *h*, we start from the “thermally untouched” body, heat it locally by the sensor in its active mode and get *ĥ*. Afterwards we determine the heat transfer coefficient *h* and/or the corresponding Nusselt number Nu from the function *h*(*ĥ*).

**Figure 3.**
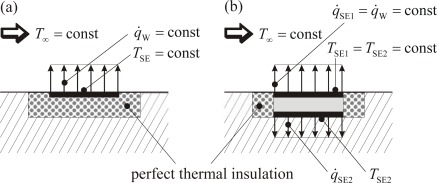
Idealized sensor configurations. (a) Single foil (SE) in a perfect thermal insulation. (b) Double foil (SE1, SE2) with a perfect counter heating by the sensitive element SE2.

Figure 3 shows a scetch of two *idealized* sensor configurations according to this concept. Both types guarantee that all dissipated energy is transferred into the fluid. In type (a) this is due to the perfect thermal insulation against the body. Nearly perfect insulation can be achieved for example by an airfilled or evacuated cavity underneath a membrane that supports the sensor element (especially a hot-film). Such sensors are realized using MEMS- and PCB- (flexible printed circuit board) technology, respectivly [11,13,16,20]. This type of sensor implementation, however, is not suitable for our purpose. Instead in type (b) a counter-heating foil (SE2) is operated in a way that it gains the same temperature as the heating foil (SE1) so that there is no heat conduction between the two foils. Insulating both foils towards the sides again guarantees that all dissipated energy of the upper foil is transferred into the fluid.

Since the foils are temperature dependent electrical resistors *R(T)*, the power *P* and the corresponding temperature *T* can be determined from the actual electric current and voltage by which the foils are operated in any mode after a *R(T)*-calibration. In the passive mode, the electric current must be reduced to a very low value in order to measure the temperature without noticeable additional heating. In the active mode (under ideal conditions scetched in Figure 3) the electric power creates the wall heat flux density *q̇*_W_ = *P*/*A*_SE_ with the acting sensor area *A*_SE_. With *q̇*_W_, *T*_W_ and *T*_∞_ the sensor specific heat transfer coefficient *ĥ* can then be determined.

## 4. Sensor Design and Manufacturing

For a sensor of type Q1 in Figure 1 special considerations concerning the design (including the choice of material) and the manufacturing process are required.

### 4.1. Design Considerations

In order to test the performance of Q1-type sensors and to verify the assumption about its modes of operation we developed a simple prototyp first and a more advanced sensor afterwards. Both sensors were designed for measurements in a two-dimensional turbulent flow field.

As a test case we chose the convective heat transfer at a (optionally heated) circular cylinder in cross flow. Once the sensor is fixed to the surface it can be brought in any position on the circumferance by rotating the cylinder around its axis as will be explained in Section 5 below.

**Figure 4.**
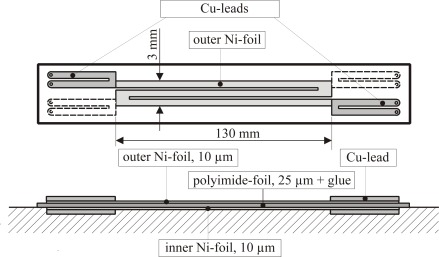
Simple sensor geometry for measurements in two-dimensional flows; the broken lines in the upper picture are the Cu-leads of the hidden Ni-foil underneath the polyimide insulation.

**Figure 5.**
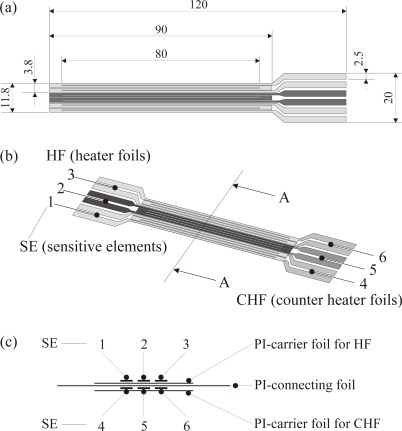
Details of the design for the advanced sensor (thin foil triple sensor). (a) photo mask. (b) combination of the numbered heater and counter heater elements. (c) cut along A-A in (b) schematic.

**Figure 6.**
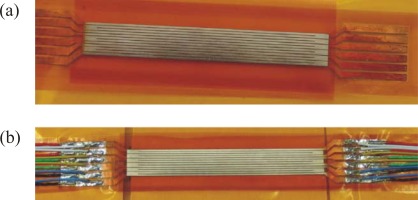
Complete laminated sensor. (a) sensor without electrical connections. (b) sensor with electrical connections.

Various aspects of the sensor design are:

- The size of the sensor is a compromise between “as small as possible for local measurements” and “as large as possible for low measurement uncertainties”. In our case this compromise was *b* = 3 mm in streamwise direction for the simple prototype according to Figure 4. Here two foils are arranged, one exactly above the other, with a thin polyimide-foil between. The design of the more advanced sensor is shown in Figure 5 and Figure 6 below.
- There should be a good contact between the sensor and the surface and only negligible interference with the flow. When sensors are flush mounted interference can be avoided completely. That, however, requires a surface intrusion and poses additional problems due to the increased heat conduction into the wall material. As an alternative, sensors should be glued to the surface. To avoid inacceptably strong interference with the flow its wall normal extend should be below *n^+^* ≈ 30, with *n^+^* being the turbulent wall coordinate (*n*^+^ = *u*_τ_*n*/*v*; $u_{\tau }=\sqrt{\tau_{W}/\rho }$: shear stress velocity; *τ*_W_: wall shear stress; *ρ*: fluid density; *ν*: kinematic viscosity [28]). In our cases an overall sensor thickness of *H* ≈ 200 μm corresponds to *H*^+^ = *u*_τ_*H*/*v* ≈ 3 at Re = 26,000.
- The material of the sensor foils should be characterized by a large temperature coefficient *α* according to equation (2). This is achieved with nickel (foils). They are well protected by an inert oxid layer. This, however, may pose problems with respect to glueing, etching and (electrically) contacting the foils.

### 4.2. Manufacturing Details

**Figure 7.**
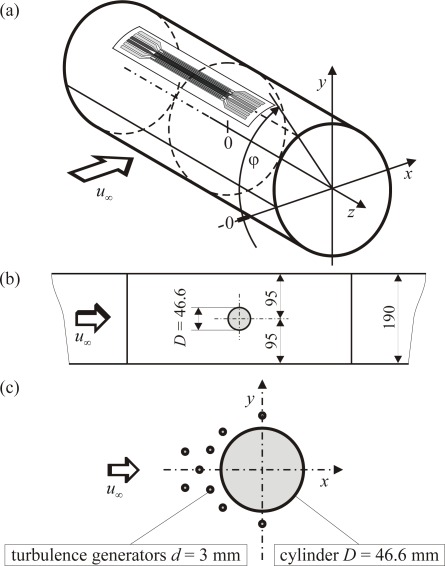
Test case geometry of the circular cylinder in cross flow. (a) Position of the triple sensor measured by φ. (b) Wind tunnel test section with the circular cylinder. (c) Upstream turbulence generators (drawn to scale).

**Figure 8.**
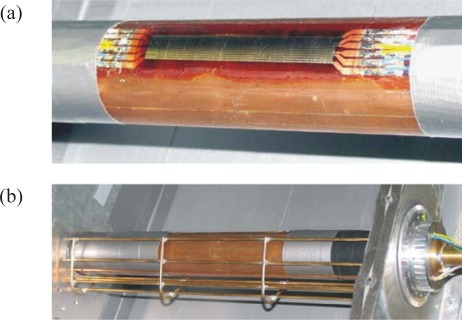
View into the wind tunnel test section. (a) Circular cylinder with mounted triple sensor; configuration for low turbulent flow field (LT). (b) Upstream mounted turbulence generators; configuration for high turbulent flow field (HT).

A manufacturing procedure was developed that allows the reliable production of film sensors with film thicknesses as low as 2 μm. We call it *foil laminating and etching technology* and regard it as a serious alternative to the often used sputtering technique. According to the experience with our technology (including many failures due to inappropriate details of the process) six steps are necessary to end up with a well-designed sensor:

1. Two Ni-foils (10 μm for the prototype in Figure 4, and 2 μm for the advanced sensor in Figures 5 – 8, respectively) are cut into roughly the size of the later sensor.
2. In a vacuum process (*p* < 10^−2^ Pa) the Ni-foils are heated until red-hot by an electrical current (25A for the 10 μm foil) for about 2 minutes. This reduces the surface oxide layer to a level that allows a subsequent copper vapour deposition of those (end) parts of the foils that will carry the electrical contacts. This is achieved by a mask that clears the area to be coated.
3. The partly copper-coated foils then are glued on two separate 25 μm carrier foils (polyimide) with a 25 μm glue layer (epoxy- resin) on one side.
4. Like an electrical printed circuit board is etched after a *photo mask* of the final metal surface is applied, the Ni-foils now take their final shape by etching away their superfluous parts. Special attention must be given to the facts that
  1. the carrier/Ni-foil combination is highly flexible
  2. adhesion of the photo resist layer is weak
  3. the etching behaviour on copper is different from that on nickel
5. Both carrier/Ni-foil combinations are glued together by a connecting foil (polyimide with glue layers on both sides) one on top of the other. The upper one now serves as heating foil, the lower one as counter-heating foil.
6. After joining the electrical contacts by soldering them to the copper coated end parts of the Ni-foils the sensor is ready for calibration in a temperature controlled calibration chamber.

With this technique first the sensor according to Figure 4 was manufactured and used on a circular cylinder in cross flow. Details of these investigations can be found in [8].

Afterwards an advanced sensor was designed which basically is the combination of three simple sensors in a row (in streamwise direction). With this advanced sensor various additional options exist and can be used to thoroughly investigate the concept of the active mode for heat transfer measurements on cold bodies. Details can be found in [29].

This advanced sensor is intruduced in the following and may again serve to illustrate the manufacturing details. Figure 5a shows the photo mask design for the etching process, the heater-counter heater foil combination is shown in Figure 5b, and pictures of the final sensor without and with electrical contacts are shown in Figure 6. This triple sensor can, for example, be used to verify the fundamental assumptions in our sensor concept, that heat transfer measurements in turbulent flows are not affected by the upstream “thermal history” of the flow. For that purposes the first two of the three double foil sensors are used as optional heaters whilst the third sensor is the actual sensor for measuring the heat transfer coefficient *h*. In chapter 6.2 such measurements will be shown.

## 5. Test Case and Calibration Procedures

### 5.1. Test Case: Circular Cylinder in Cross Flow

Figure 7a shows how the triple sensor is mounted on a circular cylinder with upstream velocity *u*_∞_ in air. The cylinder is placed in the closed test section of a wind tunnel according to Figure 7b.

The flow around a circular cylinder is especially suitable for our sensor tests. Convective heat transfer along the circumference of a cylinder varies according to the local flow conditions. Starting from front stagnation line (*φ* = 0) the flow varies from a laminar to a fully turbulent region with modifications depending on the upstream flow field characterized by the fluid velocity and its turbulence level. Different flow fields are realized by a variation of velocity and turbulence level, each in two steps.

The upstream velocities are 5 m/s and 10 m/s. The resulting Reynolds numbers, Re = *u*_∞_*D*/ν, then are Re = 13,000 and Re = 26,000, respectivly.

Different turbulence levels are a low level in the wind tunnel itself and a high level due to additional turbulence generators according to Figure 7c. Here nine metal rods are mounted parallel to the circular cylinder at positions shown in Figure 7c. These generators strongly influence the oncoming flow so that the flow around the circular cylinder is very different from the one without the turbulence generators.

The four flow fields further on are labeled as:

- (13LT) for Re = 13,000 and low turbulence
- (13HT) for Re = 13,000 and high turbulence
- (26LT) for Re = 26,000 and low turbulence
- (26HT) for Re = 26,000 and high turbulence

Figure 8 shows the circular cylinder with the double foil triple sensor attached to the surface. It can be used to measure heat transfer coefficients around the cylinder (by rotating it) in its active mode, i.e. on the “cold” cylinder.

**Figure 9.**
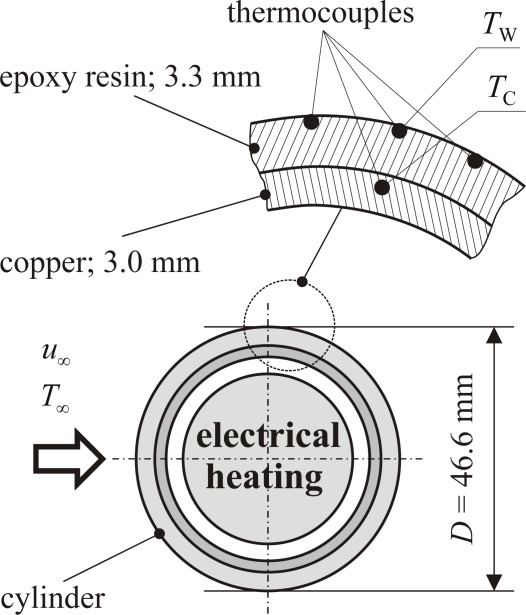
Design of the circular cylinder for local heat flux and wall temperature measurements (comparative data).

This cylinder has a special design, however, so that optionally it can also be heated and then used for the purpose of providing comparative data of the heat transfer coefficient. How this is done can be explained with the help of Figure 9.

On the heated cylinder local heat flux density and wall temperature can be simultaneously measured at fife thermocouple positions along the periphery of the heated cylinder. Due to the electrical heating inside the cylinder a certain constant temperature of the copper tube, *T*_C_, results. Wall temperatures, *T*_W_(*φ*), can be measured by the outer thermocouples at the epoxy resin surface. The local heat flux density follows from the temperature difference between the wall and the copper tube: *q̇*_W_ = (*k*/*d*)_E_(*T*_C_ – *T*_W_). Here (*k / d*)_E_ includes the thermal conductivity and the thickness of the epoxy resin, respectively. The resulting local heat transfer coefficients, $h(\varphi )=\frac{{\dot{q}}_{W}(\varphi )}{T_{W}(\varphi )-T_{\infty }}$, serve as *comparative data* for the corresponding measurements of our new thin foil sensor, see chapter 6.3 below.

### 5.2. Calibration Procedures

There are various steps in calibration of a measurement system, here for determining heat transfer values under different flow-field conditions. We suggest to introduce three categories within the final calibration procedure:

C1: Calibration of the sensor itself. Results are technical data of the sensor.
C2: Calibration with respect to systematic errors introduced by implementation of the sensor in the situation under consideration.
C3: Calibration with respect to the final physical quantity which may differ from the one that is measured directly. The targeted quantity then follows through an *equivalence relation*.

#### 5.2.1. Step C1: Calibration of the Sensor Itself

Calibration of the thin foil triple sensor (see Figure 5) corresponds to the determination of the *R*(*T*) - dependance for all six foils. Then six individual temperatures within the sensor can be determined.

This basic calibration can be performed in an air-conditioned chamber in which constant temperatures can be adjusted. For a rather small temperature band a linear calibration curve:

(9) $$R\left(T\right)=R_{0}\left[1+\alpha \left(T-T_{r}\right)\right]$$

is sufficient with *R*_0_ = *R*(*T*_0_) and *α* = (d*R /* d*T*)_0_ / *R*_0_. Here *T*_r_ is a reference temperature, which might be *T*_r_ = 0 °C, for example.

In order to also determine the three heat flux densities (in the three double foil arrangements) three values of (*k / d*) must be known. When *k* and *d* are not known exactly enough the combination (*k / d*) alternatively can be determined from the comparison of a measured heat flux density with the corresponding comparative data at a circular cylinder according to Figure 9.

Table 1 shows the *R*(*T*) - calibration data for the six foils and the three double foil combinations (*k / d*).

#### 5.2.2. Step C2: Calibration with Respect to Systematic Errors

**Figure 10.**
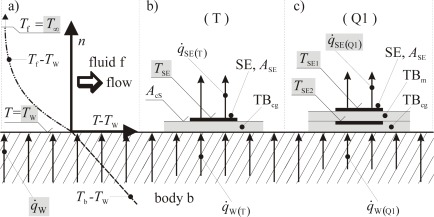
Undisturbed convective heat transfer situation and measureable values using glue-on-type sensors. a) Temperature distribution in a steady convective heat transfer according to Figure 2. b) Wall-temperature sensor glued on the surface. c) Heat-flux sensor glued on the surface.

This part of the calibration procedure accounts for systematic errors that are introduced into the measuring process by the sensor itself. For example, the wall temperature at a certain position of the wall (but without attached sensor, *T*_W_ in Figure 10a) would be slightly different from the temperature of the sensor mounted there, due to the slight modification of the physics at the measuring position by the attached sensor (*T*_SE_ in Figure 10b, *T*_SE1_ in Figure 10c). This part of the calibration process requires a thorough analysis of the systematic errors introduced by the sensor and subsequent corrections to the sensor readings.

In Figure 10a the true but unknown temperature and wall heat flux values are marked by grey shading. In Figures 10b and 10c those values are marked in the same way that can be measured by the sensor arrangement.

**Figure 11.**
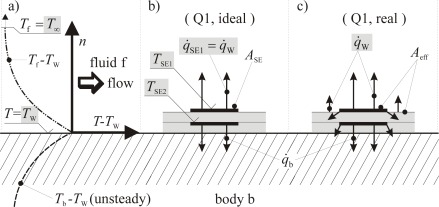
Heat transfer situation of a double foil sensor in active mode.

Figure 10b shows how *T*_SE_ ≈ *T*_W_ can be measured with a single foil arrangement, whereas Figure 10c shows a double foil sensor in passive mode with which *q̇*_SE_ ≈ *q̇*_W_ is determinded by measuring *T*_SE1_ and *T*_SE2_. It is this “≈” why a step C2-calibration is necessary. This will be further discussed for the douple foil sensor in active mode shown in Figure 11.

In Figure 11a again the temperature distribution close to the wall is shown with *T*_∞_ and *T*_W_ as true but unknown temperatures. Figure 11b shows the ideal situation (c.f. Figure 3b) in which *q̇*_SE1_ is completely transferred to the fluid on a surface *A*_SE_. Figure 11c illustrates the systematic error that occurs due to the fact that not all heat released at the upper foil is transferred into the adjacent fluid on the foil surface *A*_SE_. As a consequence the measured heat flux density cannot be determined from the heat released and the surface area *A*_SE_. In a calibration step C2 an effective area *A*_eff_ > *A*_SE_ could be determined that accounts for this. It turns out, however, that this is a difficult task (further discussed in [29]). As an alternative we combine the calibration step C2 with the subsequent step C3 and thus account for the effect of systematic errors indirectly.

#### 5.2.3. Step C3: Calibration with Respect to an Equivalence Relation

This part of the calibration procedure applies to all cases in which the targeted quantity differs from a quantity that can be directly determined from the readings of the sensor. If, for example, wall shear stress should be determined by a hot film sensor (see Figure 1; T1 active, hot film), the equivalence (Reynolds analogy) between the measured heat flux density of the mounted sensor and the wall shear stress must be found as a relation ***τ***_W_(*q̇*_W_). This can be done only when the targeted quantity in certain cases is known from independent measurements.

It turns out that our double foil sensors applied in the active mode after a basic *R(T)*-calibration do not yet measure the expected *h*-values following from *q̇*_W_, *T*_W_ and *T*_∞_ according to (7) but an equivalent “pseudo” transfer coefficient *ĥ* with *ĥ > h*.

Analysing the physics near the sensor surface in detail reveales that its surface is always part of a self-generated thermal adjustment zone after a step-change in heat flux density from *q̇*_W_ = 0 to the actual operating value. This effect can be accounted for by a sensor specific constant ***A*** which we call *thermal adjustment coefficient* or just *tac-factor*. More details about this factor can be found in [8]. The real heat transfer coefficient then is:

(10) h=Aĥ

with *ĥ* = (*P*/*S*)/(*T*_W_ – *T*_∞_), where *P* is the electrical power dissipated in the upper foil with the surface *A*_SE_ under the condition of equal temperature of this foil and the adjacent counter-heating foil. By this condition we try to approximate the ideal conditions shown in Figure 3b.

From a comparison of *ĥ* – data at the cold cylinder and the comparative *h* – data at the heated cylinder (c.f. Figure 9) we determine ***A*** = 0.277 for the double foil sensor according to Figure 4, for example. This tac-factor ***A*** is sensor specific and includes the effect of the effective transfer surface discussed in the previous sub-section (step C2-calibration).

## 6. Test Measurements, Results

With the calibrated double foil sensors heat transfer measurements can be made on cold bodies when the sensors are applied in their active mode. This will be demonstrated for the simple sensor (see Figure 4 and the advanced sensor (see Figure 5). Finally it is shown that these sensors can also be operated in their passive mode and thus act like “conventional” heat flux sensors.

### 6.1. Heat Transfer Coefficient around a Circular Cylinder (Simple Sensor)

**Figure 12.**
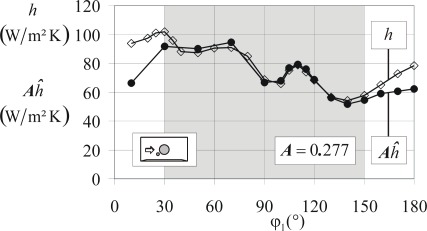
***A****ĥ* - distribution, measured with the strip sensor (Figure 4) in its active mode on the cold, unheated body compared to the conventionally measured *h* - distribution on the warm, heated body; flow field: 26HT.

Figure 12 shows ***A****ĥ* compared to the conventionally measured *h* - distribution for the Reynolds number Re = 26,000. In order to increase the turbulence level of the flow we placed a wire at φ_1_ ≈ 30° close to the cylinder surface as indicated in the insert of Figure 12. In the grey zone (30° < φ_1_ < 150°) the flow is fully turbulent and ***A****ĥ* should be equal to *h* according to the concept of our new sensor. For 150° < φ_1_ < 180°, around the downstream stagnation point in the separation region, the flow is turbulent but flow velocities are small which obviously results in larger deviations. Close to the front stagnation point (0° < φ_1_ < 30°) there is a laminar boundary layer and heat transfer physics are not determined locally as presumed in our concept.

### 6.2. Heat Transfer Coefficient around a Circular Cylinder (Advanced Sensor)

The advanced triple sensor according to Figure 5 can, for example, be used to verify the fundamental assumptions in our sensor concept, that heat transfer measurements in turbulent flows are not affected by the upstream “thermal history” of the flow. For that purpose the first two of the three double foil sensors are used as optional heaters. The third sensor then is the actual sensor for measuring *h*.

**Figure 13.**
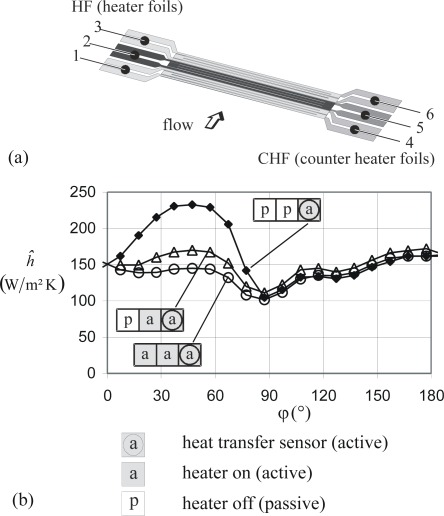
Triple sensor composed of three double foil sensors (1 ÷ 4; 2 ÷ 5; 3 ÷ 6); flow field: 26LT. (a) Design and flow direction. (b) Pseudo heat transfer coefficient *ĥ* for three different upstream thermal histories. 
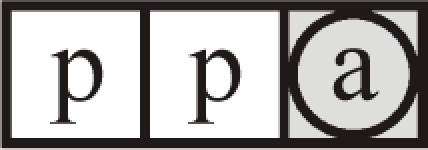
: no heating upstream; 
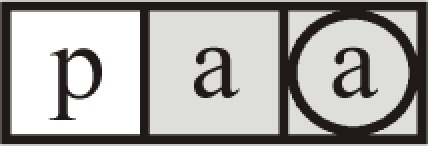
: one heating foil active upstream; 
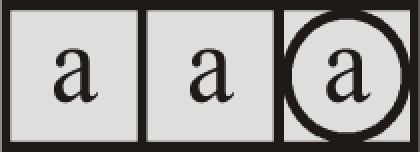
: two heating foils active upstream.

Measurements are made on the circular cylinder used before, however without turbulence promoter in front of the cylinder. Therefore, the turbulent range starts at *φ ≈* 90° now. Figure 13b shows three different cases with respect to the upstream thermal history. While there are substantial differences in *ĥ* for 0° < *φ* < 90°, they are almost absent in the turbulent range 90° < *φ* < 180°. We regard this as a corroboration of the fundamental assumption with respect to our sensor concept.

### 6.3. Double Foil Sensors in Passive Mode

**Figure 14.**
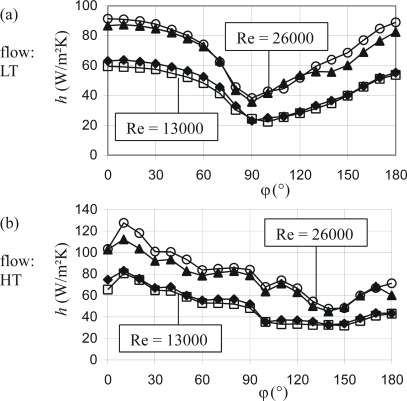
Comparative data and passively measured heat transfer coefficients. (a) black symbols ... comparative data. (b) open symbols ... passively measured data.

The two previous examples showed the double foil sensors operated in their active mode, c.f. Figure 1. They can, however, also be used in a passive mode in which they immediately measure a heat flux density only, c.f. again Figure 1. With the additional information about the free stream temperature, *T*_∞_, the heat transfer coefficient, *h* = *q̇*_W_/(*T*_W_ – *T*_∞_), can be determined. In this mode, calibration with respect to (*k / d*) would be required. This can be circumvented by the indirect determination of (*k / d*) for the sensor through adjusting *h* – results once to those of the comparative data (keeping this (*k / d*) from thereon). Figure 14 shows that then in all four cases (13LT, 13HT, 26LT, 26HT) there is a good match between both methods of measurement.

## 7. Error Analysis

The quantities to measure are the temperatures *T*_SE_ and the dissipated electrical powers *P*_SE_ = *Q̇*_SE_ = *q̇*_SE_*A*_SE_ of the SE (see Figures 10 and 11). The temperatures *T*_SE_ follow from the measured resistances *R*_SE_ taking into accound their calibration curves, c.f. equations (3) and (6). *R*_SE_ and *P*_SE_ values are

(11) $$\begin{array}{cc} R_{\text{SE}}=\left(U_{\text{SE}}/U_{V}\right)R_{V}; & P_{\text{SE}}=\left(U_{\text{SE}}U_{V}\right)/R_{V} \end{array}$$

based on the voltage drops along the resistances *R*_SE_ and their corresponding serial resistors *R*_V_.

The uncertainties of *T*_SE_ and *P*_SE_ then are:

(12) $$\begin{array}{cc} \Delta T=\frac{R}{{\alpha R}_{0}}\left[\frac{{\Delta U}_{V}}{U_{V}}+\frac{{\Delta U}_{\text{SE}}}{U_{\text{SE}}}+\frac{{\Delta R}_{V}}{R_{V}}\right]; & \Delta P=P_{\text{SE}}\left[\frac{{\Delta U}_{V}}{U_{V}}+\frac{{\Delta U}_{\text{SE}}}{U_{\text{SE}}}+\frac{{\Delta R}_{V}}{R_{V}}\right] \end{array}$$

The uncertainties of the voltage depend on the voltage itself and the properties of the voltmeters used: *ΔU / U = f(U)*. Uncertainties of the serial resistors *R*_V_ are systematic errors, which from measurements with a Wheatstone bridge are *ΔR*_V_ / *R*_V_ ≈ 10^−3^. This uncertainty can be neglected when measurements are compared that only differ in certain parameter values.

**Figure 15.**
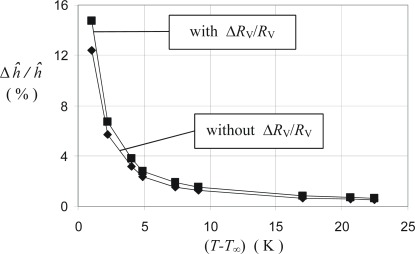
Relative uncertainty for *ĥ* - measurements with the DFS 2 ÷ 5 shown in Figure 5.

For the main quantity of interest, the actively measured (pseudo) heat transfer coefficient *ĥ* we get the uncertainty diagram Figure 15. Since *ĥ* - data are determined with temperature differences (*T – T*_∞_) between 5K and 13K relative uncertainties are always Δ*ĥ/ĥ* < 3%.

Uncertainties *ΔT*_SE_ are 0.02 K ≤ *ΔT*_SE_ ≤ 0.06 K. These values correspond with the voltage range 5 V ≥ *U*_SE_ ≥ 0.03 V. The uncertainty of *q̇*_SE_ (passive mode, see Figures 10c and 12) are (Δ*q̇*_SE_/*q̇*_SE_) ≤ 20%. This uncertainty is rather large due to the large values of (*k / d*), see Table 1.

## 8. Conclusions

We demonstrated how a conventional double foil sensor can be operated in an active mode in order to measure local heat transfer coefficients. Various sensor designs are possible, for example square shaped sensors for measurements in flow situations that are not two-dimensional (like the one we used here). With the basic concept approved we would like to encourage other groups to adopt the idea and realize it within their own context of heat transfer problems.

A special challenge certainly would be to miniaturize sensors according to the concept presented thus getting into the field of MEMS technology. One of the inherent advantages would be the reduction in space and time scales of such sensors. That would open a wide variety of applications in various fields with heat transfer problems.

## Figures and Tables

**Table 1.** C1-calibration data for the thin foil triple sensor (TFTS).

**Electrical sensor data**				**Thermal sensor data**	
TFTS SE	*R*_SE0_ [Ω]	*αR*_SE0_ [mΩ/K]	*α* [10^−3^/K]	DFS	(*k / d*) [W/m^2^K]
1	16.31	78.49	4.81	1 ÷ 4	800
2	16.18	77.96	4.82		
3	16.08	77.64	4.83	2 ÷ 5	705
4	15.34	77.02	5.02		
5	15.42	78.16	5.07	3 ÷ 6	705
6	15.43	77.94	5.05		

## Nomenclature

***A***: thermal adjustment coefficient (-)
*A*: area (mm^2^)
*b*: width (mm)
*C*: coefficient (-)
*D*: diameter (mm)
*d*: diameter, thickness (mm)
*H*: sensor thickness (μm)
*h*, *ĥ*: heat transfer coefficient (W/m^2^K)
*k*: thermal conductivity (W/mK)
*L*: characteristic length of a heat transferring sensor or body (mm)
*l*: length (mm)
*n*: wall coordinate (μm)
*n*^+^: turbulent wall coordinate (-)
Nu: Nusselt number (-)
*P*: electrical power (mW)
*p*: pressure (Pa)
*q̇*: heat flux density (W/m^2^)
*R*: electrical resistance (Ω)
Re: Reynolds number (-)
*S*: sensitivity of the TSE (Ω/K)
*T*: temperature (°C)
*U*: voltage (V)
*u*: fluid velocity (m/s)
*u*_τ_: shear stress velocity (m/s)

**Greek symbols**
*α*: temperature coefficient of electrical resistance (1/K)
*φ*: angle-position of the sensor, glued on the circular cylinder (°)
*ν*: kinematic viscosity (m^2^/s)
*ρ*: fluid density (kg/m^3^)
*ρ*_e_: electrical resistivity (Ωm)
*τ*_W_: wall shear stress (N/m^2^)

**Subscripts**
0: baseline
∞: undisturbed fluid
b: body
c: cross section
C: copper tube
cg: carrier and glue
cs: complete sensor
e: electrical
E: epoxy resin
eff: effective
f: fluid
l: lower surface
m: measure
r: reference
R: resistance
SE: sensitive element
V: placed in series
W: wall

**Abbreviations**
CHF: counter heater foil
DFS: double foil sensor
HE: heating element
HF: heater foil
HT: high turbulence
LT: low turbulence
MEMS: microelectromechanical system
PCB: printed circuit board
PI: polyimide
RTS: resistance temperature sensor
SE: sensitive element
TSE: temperature sensitive element
TFTS: thin foil triple sensor
